# Animal Functional Magnetic Resonance Imaging: Trends and Path Toward Standardization

**DOI:** 10.3389/fninf.2019.00078

**Published:** 2020-01-22

**Authors:** Francesca Mandino, Domenic H. Cerri, Clement M. Garin, Milou Straathof, Geralda A. F. van Tilborg, M. Mallar Chakravarty, Marc Dhenain, Rick M. Dijkhuizen, Alessandro Gozzi, Andreas Hess, Shella D. Keilholz, Jason P. Lerch, Yen-Yu Ian Shih, Joanes Grandjean

**Affiliations:** ^1^Singapore Bioimaging Consortium, Agency for Science, Technology and Research, Singapore, Singapore; ^2^Faculty of Biology, Medicine and Health, The University of Manchester, Manchester, United Kingdom; ^3^Center for Animal MRI, Department of Neurology, Biomedical Research Imaging Center, The University of North Carolina at Chapel Hill, Chapel Hill, NC, United States; ^4^Direction de la Recherche Fondamentale, MIRCen, Institut de Biologie François Jacob, Commissariat à l’Énergie Atomique et aux Énergies Alternatives, Fontenay-aux-Roses, France; ^5^Neurodegenerative Diseases Laboratory, Centre National de la Recherche Scientifique, UMR 9199, Université Paris-Sud, Université Paris-Saclay, Fontenay-aux-Roses, France; ^6^Biomedical MR Imaging and Spectroscopy Group, Center for Image Sciences, University Medical Center Utrecht, Utrecht University, Utrecht, Netherlands; ^7^Department of Psychiatry, Douglas Mental Health University Institute, McGill University, Montreal, QC, Canada; ^8^Department of Biological and Biomedical Engineering, Douglas Mental Health University Institute, McGill University, Montreal, QC, Canada; ^9^Functional Neuroimaging Laboratory, Istituto Italiano di Tecnologia, Centre for Neuroscience and Cognitive Systems @ UNITN, Rovereto, Italy; ^10^Institute of Experimental and Clinical Pharmacology and Toxicology, Friedrich–Alexander University Erlangen–Nürnberg, Erlangen, Germany; ^11^Department of Biomedical Engineering, Georgia Tech, Emory University, Atlanta, GA, United States; ^12^Hospital for Sick Children, Department of Medical Biophysics, University of Toronto, Toronto, ON, Canada; ^13^Wellcome Centre for Integrative NeuroImaging, University of Oxford, Oxford, United Kingdom; ^14^Department of Radiology and Nuclear Medicine, Donders Institute for Brain, Cognition, and Behaviour, Donders Institute, Radboud University Medical Center, Nijmegen, Netherlands

**Keywords:** resting-state, rodent, non-human primate, optogenetics, DREADD

## Abstract

Animal whole-brain functional magnetic resonance imaging (fMRI) provides a non-invasive window into brain activity. A collection of associated methods aims to replicate observations made in humans and to identify the mechanisms underlying the distributed neuronal activity in the healthy and disordered brain. Animal fMRI studies have developed rapidly over the past years, fueled by the development of resting-state fMRI connectivity and genetically encoded neuromodulatory tools. Yet, comparisons between sites remain hampered by lack of standardization. Recently, we highlighted that mouse resting-state functional connectivity converges across centers, although large discrepancies in sensitivity and specificity remained. Here, we explore past and present trends within the animal fMRI community and highlight critical aspects in study design, data acquisition, and post-processing operations, that may affect the results and influence the comparability between studies. We also suggest practices aimed to promote the adoption of standards within the community and improve between-lab reproducibility. The implementation of standardized animal neuroimaging protocols will facilitate animal population imaging efforts as well as meta-analysis and replication studies, the gold standards in evidence-based science.

## Introduction

A detailed understanding of the mammalian brain structure and function is one of the greatest challenges of modern neuroscience. Approaching the complexity of the organ and the levels of organization of neuronal circuits across several orders of magnitudes, both spatially and temporally, requires the collective scientific efforts from multiple teams across several disciplines. Neuroimaging, especially by means of magnetic resonance imaging (MRI), is playing a preponderant role in mapping the human and animal brain, due to its non-invasiveness, excellent soft-tissue contrast, and multiple readouts. The human neuroimaging research has accelerated over the past decade, fueled by numerous discoveries about brain structure and function and its relation to disorders. In turn, this has led to population imaging efforts aimed to describe variations in brain structure and function, and their relation to behavioral traits, genetic polymorphisms, and pathology. For instance, since its original description in 1995 (Biswal et al., 1995), resting-state functional connectivity (RS-FC) has been at the center of numerous population imaging initiatives, such as the 1,000 Functional Connectomes Project (Biswal et al., 2010), the WU-Minn Human Connectome Project (Van Essen and Ugurbil, 2012; Van Essen et al., 2013), and the UK Biobank (Miller et al., 2016). In addition to providing an important baseline of healthy cohorts, these initiatives are complemented with population imaging dedicated to specific psychiatric and neurological disorders, such as the Alzheimer’s Disease Neuroimaging Initiative (Petersen et al., 2010; Weiner et al., 2012), the Autism Brain Imaging Data Exchange (Di Martino et al., 2014), or Attention-Deficit Hyperactivity Disorder (HD-200 Consortium, 2012). Collectively, these resources have significantly advanced our understanding of neuro- and psychopathologies, as well as providing an understanding of disorder spectrums at a population level.

**FIGURE 1:**
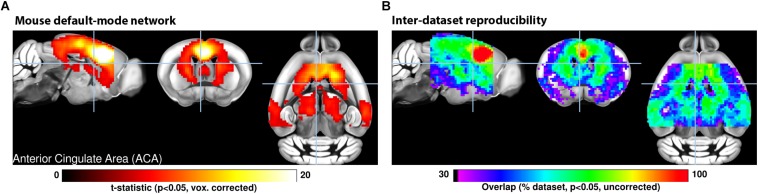
**(A)** A seed-based analysis of the anterior cingulate area in 98 resting-state fMRI scans reveals the topological distribution of the mouse default-mode network. The regions co-activating with the seed include the dorsal striatum, dorsal thalamus, retrosplenial, and posterior parietal areas. **(B)** The reproducibility of the default-mode network was assessed in 17 independent datasets consisting of 15 scans each. Overlapping one-sample *t*-test maps are summarized in a color-coded overlay. 12/17 datasets present converging topological features, the remaining five failed to present evidence of distal connectivity relative to the seed. Adapted with permission from Grandjean et al. (2019a).

In contrast to the above, functional neuroimaging studies in animals have remained mostly confined to single centers, often relying on lab-specific acquisition and processing protocols. There has been little pressure toward standardization within the community, and results from different centers have remained inherently difficult to compare, due to discrepancies related to animal housing and preparation, recording hardware, and analysis methodologies. It is now emerging that these preparation divergences are at the stem of a number of dissensions within the animal functional neuroimaging community, such as the nature of unilateral vs. bilateral resting-state networks (RSN) in mice (Jonckers et al., 2011; Grandjean et al., 2014; Mechling et al., 2014; Sforazzini et al., 2014), the bilateral BOLD response to non-noxious paw electrical stimulation in mice (Bosshard et al., 2010; Schroeter et al., 2014; Shim et al., 2018), the indirect artifacts emerging in optogenetics fMRI (ofMRI) through either heating or vascular photoactivation (Christie et al., 2013; Rungta et al., 2017; Schmid et al., 2017), or the spatial extent of distributed networks of translational relevance, such as the rodent “default mode network” (DMN) reviewed in Gozzi and Schwarz (2016). Only recently did efforts emerge to combine and compare structural and/or functional MRI from multiple centers in monkeys (Milham et al., 2018) and in mice (Figure 1; Grandjean et al., 2019a). These initial studies provide solid grounds for the development of replication studies, meta-analyses, and multi-center consortia, the gold standards in evidence-based science.

Presently, we aim to describe the current trends in the field and to examine how these impact the results and their comparability with the rest of the literature. While recommendations to enhance reproducibility exists for human neuroimaging (Poldrack et al., 2008), a large number of acquisition and data processing aspects remain specific to animal imaging. We systematically assessed the animal fMRI literature for data acquisition and analysis procedures to provide an overview of the collective directions taken within the animal imaging community. We then reviewed the major considerations taking place in the study design, and how these impact results and their interpretability. Finally, we use this information to provide a road map toward the adoption of standards that will enable animal population studies to inform on the functional mammalian brain.

## Methods

We searched the Pubmed database^1^ on February 11, 2019 for the terms “functional magnetic resonance imaging,” “functional MRI,” or “fMRI” within the abstract or title, excluding studies in human and reviews, from 1990 onward, using the following command. *“Search ((fMRI[Title/Abstract]) OR functional MRI[Title/Abstract]) OR functional magnetic resonance imaging[Title/Abstract] Sort by: Best Match Filters: Abstract; Publication date from 1990/01/01 to 2019/12/31; Other Animals.”* The query returned 2279 entries. The title and abstract from these were manually screened to exclude studies that did not contain primary research using MRI to assess brain function in animals. In total, 868 research article were considered relevant and could be readily accessed. We recorded the type of study: resting-state or paradigm free RS-FC recordings, pharmacological-evoked, opto-/chemogenetic neuromodulation, deep-brain stimulation (DBS), or stimulus-evoked (including blocks- or events-related designs with sensory stimulation, gas challenge, etc.). We recorded animals species, including strain, gender (male, female, both, N/A), number of animals used, animal preparation (awake, anesthetized free-breathing, anesthetized ventilated), anesthetic used for maintenance during fMRI, field strength, fMRI sequence and contrast, pre-processing softwares, and noted if the datasets were made available by the authors or in online repositories. The resulting table is made available in the Supplementary Material.

## Results and Discussion

### Experimental Design

Animal fMRI presents the opportunity for new and creative directions in study design, but care must be taken to ensure that experimental changes in the fMRI signal are sufficiently robust for detection and that results are not contaminated by procedural artifacts. Here we highlight evidence supporting standards and reporting strategies to optimize data quality, interpretation, and reproducibility for several common animal fMRI paradigms.

#### Stimulus-Evoked fMRI

In animal studies, stimulus-evoked fMRI usually refers to externally applied stimuli during fMRI (e.g., electrical forepaw stimulation), but many principles of study design can be applied to internally delivered stimuli as well, such as with deep-brain stimulation (DBS) and optogenetics. Stimuli can be applied in a block or event-related design. The former alternates between regular stimulation and no-stimulation conditions, while the latter uses brief stimuli presented at varying intervals (Amaro and Barker, 2006). Block designs are best suited to test frequency-related responses and enhance detection power, while event-related designs are best for determining accurate response-time courses and/or frequency-independent functional connectivity (Amaro and Barker, 2006; Van der Linden et al., 2007; Maus and van Breukelen, 2013; Allen et al., 2015; Schlegel et al., 2015; Soares et al., 2016).

Stimulus frequency has a large influence on stimulus-evoked fMRI results. In general, higher frequencies will increase the stimulus input per unit time, thus potentially boosting signal and ability to detect evoked responses (Amaro and Barker, 2006; Kim et al., 2010; Maus and van Breukelen, 2013), but excessive electrical or optical stimulation can cause tissue damage (Kiyatkin, 2007; Lai et al., 2015; Acker et al., 2016; Cogan et al., 2016), heating and related artifacts (Zeuthen, 1978; Kiyatkin, 2007; Cardin et al., 2010; Christie et al., 2013; Lai et al., 2015; Stujenske et al., 2015; Acker et al., 2016), and non-specific effects (Tuor et al., 2002; Christie et al., 2013; Schroeter et al., 2014; Shih et al., 2014; Schlegel et al., 2015; Rungta et al., 2017). Stimuli may also change basic physiology and therefore alter the fMRI response (Tuor et al., 2002; Ray et al., 2011; Tsubota et al., 2012; Li et al., 2013; Schroeter et al., 2014; Shih et al., 2014; Reimann C. et al., 2018), thereby occluding signal from the stimulus itself. These findings highlight the importance of carefully monitoring physiology (see below) and establishing frequency-response curves for the stimuli of choice.

#### Functional Connectivity MRI

**FIGURE 2:**
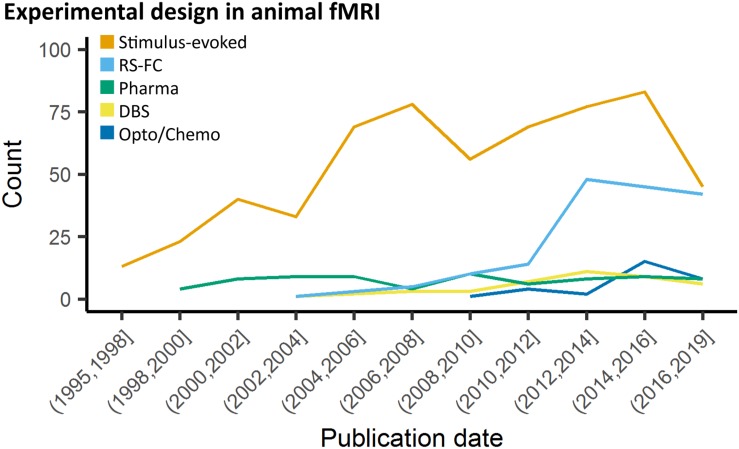
Study design in animal fMRI over time. Stimulus-evoked fMRI (events or blocks related) remain the major component within animal literature. From 2006 and 2010, resting-state fMRI and opto-/chemogenetic fMRI, respectively, have represented an increasing proportion of the animal fMRI studies.

Animal fMRI data acquired in the absence of stimulation or modulation, RS-FC, is commonly used to probe synchronization of spontaneously fluctuating signals between combinations of anatomically, functionally, or procedurally defined brain regions (Lowe et al., 2000; Lu et al., 2007; Zhao et al., 2008; van Meer et al., 2010, 2012; Lu and Stein, 2014; Pan et al., 2015; Guadagno et al., 2018; Grandjean et al., 2019a). The use of RS-FC in animal models has rapidly increased over the past decade (Figure 2). To collect the most robust and interpretable RS-FC data, a few principles have been proposed. Recent evidence suggests that brain network components exhibit non-stationary properties (Hutchison et al., 2013a; Keilholz et al., 2013; Liu and Duyn, 2013; Liang et al., 2015a; Pan et al., 2015; Gutierrez-Barragan et al., 2018), therefore repetition time should be sufficiently short (e.g., 1 s) to properly sampled the fluctuations and to detect these changes, and scan length should produce enough frames (a minimum of about 300) to account for a large number of temporal clusters (Majeed et al., 2011; Hutchison et al., 2013b; Jonckers et al., 2015). Critical aspects for such analyses are detailed in a later section. Furthermore, if brain modulation/stimulation is included, additional time should be added during the transition periods to and from resting-state to allow for stable connectivity, and subsequent resting periods following each manipulation should be grouped separately to account for potential neuroadaptations (Pawela et al., 2008; Zhao et al., 2008; Jonckers et al., 2015; Albaugh et al., 2016; Chan et al., 2017; Decot et al., 2017; Chen et al., 2018). Importantly, due to the nature of the signal fluctuations on which RS-FC relies, special care must be ensured with regard to physiology and anesthesia to ensure maximal detection. The effects of animal preparations are further discussed below.

#### Optogenetics

Many recent stimulus-evoked animal fMRI studies take advantage of the readily MR-compatible optogenetics toolkit (Figure 2; Desai et al., 2011; Abe et al., 2012; Scott and Murphy, 2012; Kahn et al., 2013; Iordanova et al., 2015; Lemieux et al., 2015; Liang et al., 2015b; Takata et al., 2015; Weitz et al., 2015; Albaugh et al., 2016; Chai et al., 2016; Ryali et al., 2016; Yu et al., 2016; Hinz et al., 2017; Lohani et al., 2017; Albers et al., 2018; Brocka et al., 2018; Choe et al., 2018; Leong et al., 2018; Grandjean et al., 2019b). Optogenetics allows for robust stimulation of specific cellular and/or anatomical populations (Zhang et al., 2010; Fenno et al., 2011; Boyden, 2015; Deisseroth, 2015; Griessner et al., 2018), but despite these advantages this relatively new technique adds layers of complexity over DBS, thereby requiring more rigorous methodology and additional controls.

The light-activated channels/pumps expressed in optogenetics, also known as “opsins,” provide a great deal of experimental flexibility (Fenno et al., 2011; Deisseroth, 2015; Guru et al., 2015). There are several opsins to choose from for optical excitation of cells, including the commonly used ChR2 (Nagel et al., 2003; Boyden et al., 2005; Zhang et al., 2006; Atasoy et al., 2008; Cardin et al., 2010) variants activated by penetrating red-shifted light (Zhang et al., 2008; Lin et al., 2013; Klapoetke et al., 2014) and ultra-fast variants capable of frequencies up to 200 Hz (Lin et al., 2009; Gunaydin et al., 2010; Hight et al., 2015). If stable excitation over even longer periods is required in fMRI, issues with a continuous light application can be avoided by using step-function opsins which are temporarily activated by a single pulse of light (Berndt et al., 2009; Ferenczi et al., 2016). Notably, there are also several opsins for cellular inhibition (Zhang et al., 2007; Berndt et al., 2014; Chuong et al., 2014), but their application for fMRI is limited as they require longer periods of illumination prone to heat-related artifacts, and anesthetized or sedated animals have low baseline levels of activity (Lahti et al., 1999; Brevard et al., 2003; Sicard et al., 2003).

Injection of viral constructs or expression of foreign genes can potentially change brain function (Liu et al., 1999; Klein et al., 2006; Zimmermann et al., 2008; Lin, 2011; Miyashita et al., 2013), and light can induce heating and related MRI artifacts, tissue damage, and non-specific effects (Elias et al., 1987; Christie et al., 2013; Stujenske et al., 2015; Schmid et al., 2016; Rungta et al., 2017) thus it is critical to characterize opsin expression and activation of the light source with light delivery to empty-vector (e.g., EYFP) controls. It follows that histological confirmation of fiber placement and construct co-localization with targeted promoters is required (Bernstein and Boyden, 2011; Witten et al., 2011; Madisen et al., 2012; Zeng and Madisen, 2012; Allen et al., 2015; Gompf et al., 2015; Lin et al., 2016; Decot et al., 2017). In addition, given the spatial nature of fMRI, the reporting of single-point measurements of light power should be avoided in favor of irradiance (mW/mm^2^; Aravanis et al., 2007; Huber et al., 2008; Kahn et al., 2011; Yizhar et al., 2011; Schmid et al., 2017). Finally, light stimulation at frequencies at or below 20 Hz can produce a visual response by activating the visual-related network, requiring light masking or careful control comparison to view experimental effects (Ferenczi et al., 2016; Lin et al., 2016; Decot et al., 2017; Schmid et al., 2017).

#### Chemogenetics

Chemogenetics, initially termed “pharmacogenetics,” utilizes pharmacologically inert ligands to stimulate genetically encoded designer receptors, with the aim to produce drug-like sustained activation or inhibition of specific neuronal populations. Initial attempts to combine this approach with fMRI have involved the regional re-expression of pharmacologically targetable endogenous G-coupled protein receptors (e.g., Htr1a, Gozzi et al., 2012). The recent development of a modular set of evolved G protein-coupled receptors, termed Designer Receptors Exclusively Activated by Designer Drugs (DREADDs) has greatly expanded the capabilities of this approach (Armbruster et al., 2007; Alexander et al., 2009; Lee et al., 2014; English and Roth, 2015; Roth, 2016; Sciolino et al., 2016; Smith et al., 2016; Zhu et al., 2016; Aldrin-Kirk et al., 2018). Like optogenetics, chemogenetics is readily MRI compatible (Giorgi et al., 2017; Roelofs et al., 2017; Chen et al., 2018; Griessner et al., 2018; Markicevic et al., 2018). Despite its potential, there is, however, an ongoing debate about the specificity of chemogenetics ligands both in neurobehavioral studies (MacLaren et al., 2016; Gomez et al., 2017; Mahler and Aston-Jones, 2018; Manvich et al., 2018) and in chemo-fMRI applications (Giorgi et al., 2017), thereby requiring rigorous methodology to control for potential off-target effects.

Both hM3Dq and hM4Di DREADDs are classically activated with infusion of the effector clozapine-N-oxide (CNO) (Armbruster et al., 2007; Alexander et al., 2009; Roth, 2016; Smith et al., 2016; Giorgi et al., 2017; Markicevic et al., 2018), but new evidence suggests that CNO does not cross the blood-brain barrier and instead is back-metabolized *in vivo* into its precursor, clozapine (Gomez et al., 2017; Mahler and Aston-Jones, 2018; Manvich et al., 2018). Importantly, unlike CNO, clozapine is a psychoactive drug, that possesses an affinity for many endogenous receptors. As a result, the use of high CNO doses may result in a plethora of undesirable off-target effects (Ashby and Wang, 1996; Selent et al., 2008; MacLaren et al., 2016; Roth, 2016), including unspecific fMRI response (Giorgi et al., 2017). Overall, it is apparent that chemogenetics effects cannot be interpreted without proper non-DREADD expressing controls. Specifically, the effect of effector administration should be compared between DREADD expressing, and non-DREADD expressing animals and/or hemispheres. Finally, as with optogenetics, validation of DREADD expression and co-localization with target promoters is essential for data interpretation (Farrell et al., 2013; Smith et al., 2016; Giorgi et al., 2017; Gomez et al., 2017; Roelofs et al., 2017; Aldrin-Kirk et al., 2018; Chen et al., 2018; Markicevic et al., 2018).

#### Pharmacological fMRI

Modulating the brain with pharmacological agents during animal fMRI has a wide variety of traditional applications such as studying the global effects of compounds and their target neurotransmitter systems (Mueggler et al., 2001; Shah et al., 2004; Ferrari et al., 2012; Razoux et al., 2013; van der Marel et al., 2013; Jonckers et al., 2015). This approach does not require surgical methods, and is apt for identifying global or regional changes in function associated with new or existing drug therapies for neurotransmitter-related brain disorders (Leslie and James, 2000; Martin and Sibson, 2008; Canese et al., 2011; Bifone and Gozzi, 2012; Klomp et al., 2012; Minzenberg, 2012; Medhi et al., 2014), or to map the effect of exogenously administered neuromodulators. In addition, pharmacological challenges can be used to probe how targets and neurotransmitter systems modulate BOLD responses evoked by other stimuli or pharmacological agents (Marota et al., 2000; Hess et al., 2007; Schwarz et al., 2007; Knabl et al., 2008; Rauch et al., 2008; Shih et al., 2012a; Squillace et al., 2014; Shah et al., 2016; Decot et al., 2017; Bruinsma et al., 2018; Griessner et al., 2018). However, functional imaging with pharmacological agents may not be ideal for dynamic or repetitive studies as effects are dependent on diffusion and receptor kinetics (Steward et al., 2005; Ferris et al., 2006; Mandeville et al., 2013; Bruinsma et al., 2018), and subject to receptor desensitization and downregulation (Chen et al., 1999; Arey, 2014; Berg and Clarke, 2018); which in some instances may be species-specific (Knabl et al., 2008).

It is important to consider dose-response effects and the pharmacokinetics of each drug used in the experimental design. Ideally several doses of drug, and sufficiently long time series should be included in order to interpret the results according to dose-response and absorption/elimination functions (Leslie and James, 2000; Marota et al., 2000; Mueggler et al., 2001; Steward et al., 2005; Ferris et al., 2006; Rauch et al., 2008; Jenkins, 2012; Minzenberg, 2012; Jonckers et al., 2015; Shah et al., 2015; Bruinsma et al., 2018). Indeed, many pharmacological agents have known systemic effects which can influence animal physiology and the BOLD signal (Shah et al., 2004; Wang et al., 2006; Martin and Sibson, 2008; Ferrari et al., 2012; Klomp et al., 2012), and some drugs have direct effects on the vascular endothelium in the brain, which could alter properties of the hemodynamic response (Luo et al., 2003; Gozzi et al., 2007; Shih et al., 2012b). It is imperative to closely control and monitor animal physiology, and use appropriate doses in order to control for unwanted side effects. Importantly, vehicle controls are necessary for any pharmacological fMRI study, as increased blood flow/volume and increased blood pressure from systemic infusions can alter the MRI signal (Kalisch et al., 2001; Tuor et al., 2002; Gozzi et al., 2007; Reimann H. M. et al., 2018).

### Species, Sample Size, and Gender Distribution

**FIGURE 3:**
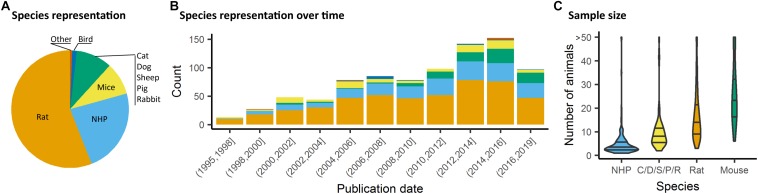
Species distribution and sample size. **(A)** Animal representation in the documented studies. **(B)** Animal species occurrence in the literature over time. Rats and non-human primate (NHP) represent the major species used, however, since 2008, mice have been used in a growing proportion of animal fMRI studies. **(C)** Number of animals used per fMRI study irrespective of number of groups or classes. NHP studies are carried out with fewer animals (Percentiles_25, 50, 75_ = [2, 3, 5]), whereas studies involving mice involved larger number of animals (Percentiles_25, 50, 75_ = [17, 24, 34]).

We assessed studies performed using animals, i.e., all species except homo sapiens. The rat and specifically the Sprague–Dawley strain was the most common species and strain used in fMRI studies, representing 55% of the total studies considered presently (Figures 3A,B). Non-human primate (NHP) studies were second and mostly relied on the macaques (23%). Studies involving medium-sized domestic mammals (cats, dogs, sheeps, pigs, and rabbits) presented 9% of the total literature considered. Studies on males (54%) had a higher incidence than studies in females (14%). A sizable number of studies (22%) omitted to specify the gender. This gender bias reflects a greater trend found throughout neuroscience and other biomedical disciplines (Beery and Zucker, 2011) and should require a greater consideration within the animal neuroimaging community. Finally, the total number of animals was assessed within the studies considered. It should be noted that this was done irrespective of the number of groups. There, we found that nearly half the studies were carried out on ten or fewer subjects (Figure 3C). This was particularly marked in studies with NHP (Percentiles 25, 50, 75 = [2, 3, 5]). While sample size depends on the goals of each study and appropriate power calculation, it remains unclear how group sizes were determined in most of these studies. The small group sizes reported here are consistent with general trends in neuroscience toward underpowered studies. Button et al. (2013) estimated that the median power level in neuroscience was at 21%. Hence these trends need to be carefully taken into consideration in the initial stages of study design so that the required animals are used to their full potential.

The wide range of experimental animals available for research offers unique opportunities to study evolutionary trends on distributed neuronal networks. To date, however, interspecies comparisons have remained a difficult task. fMRI has provided numerous descriptions of the network organization in mammals. Specifically, RSNs have been mainly studied in mammals to develop translational models of human diseases and to understand the mechanisms underlying their functional alterations. RSNs’ organization has been described in numerous mammalian species (usually under anesthesia) including rodents (Hutchison et al., 2010; Jonckers et al., 2011; Sforazzini et al., 2014; Grandjean et al., 2017b), ferrets (Zhou et al., 2016), rabbits (Schroeder et al., 2016), dogs (Kyathanahally et al., 2015), prairie vole (Ortiz et al., 2018), and NHP (Vincent et al., 2007; Hutchison et al., 2010; Mantini et al., 2011; Belcher et al., 2013). Particularly active at rest, one of the most widely investigated networks is the DMN (Raichle, 2015; Buckner and DiNicola, 2019). This network comprises distributed polymodal cortices that are thought to be involved in memory consolidation and higher cognitive functions. Homologs of the human DMN (Raichle et al., 2001) have been identified in a variety of species including NHP (Vincent et al., 2007; Mantini et al., 2011), rats (Lu et al., 2012), mice (Sforazzini et al., 2014; Stafford et al., 2014) and rabbits (Schroeder et al., 2016). The hypothesis of two separated DMNs (anterior and a posterior) has been evoked in dogs (Kyathanahally et al., 2015) and ferrets (Zhou et al., 2016).

The description of each species’ functional architectures has been based on a variety of acquisitions, analyses, and anesthesia or awake protocols. This lack of interspecies standardization is often justified by the variety of brain sizes, different response to anesthesia, and anatomical organizations observed within mammals. Throughout evolution, brain regions could have duplicated, fused, reorganized or expanded (Hutchison and Everling, 2012). A few studies have compared the connectivity between different species and with similar approaches. Using ICA, Jonckers et al. found that the extracted components, i.e., functional network regions, were more unilateral in mice compared to rats (Jonckers et al., 2011), however, this effect failed to be replicated in numerous follow-up studies in mice (e.g., Grandjean et al., 2014; Sforazzini et al., 2014). In mouse lemur primates and humans, the cortical large-scale networks repertoire presents important similarities but the regional organization into networks highlighted compositional and structural divergences (Garin et al., 2019). Strong interhemispheric functional connectivity (FC) between homotopic regions has been consistently observed in humans and primates suggesting a phylogenetically preserved mammalian characteristic (Hutchison and Everling, 2012). However, lateralized networks (i.e., fronto-parietal resting-state network) remain a phenomenon which has only been demonstrated in humans. According to the few comparative studies on mammals functional organization, humans seem to display the strongest variety of functional networks. The complexity and diversity of the animal behaviors are probably related to this large repertoire of networks. This complexity is also reflected by the white matter fiber tracts network (Nadkarni et al., 2018). Moreover, direct evidence is in favor of a close relationship between the structural and functional organization in humans (Damoiseaux and Greicius, 2009), in primates (Miranda-Dominguez et al., 2014) and in mice (Stafford et al., 2014; Grandjean et al., 2017b). However, a recent systematic review showed that structure-function correlations in mammalian brains depend on the connectivity measures, which differ across methods and scales (Straathof et al., 2019). The structure-function correspondence observed in multiple species is an important step in favor of the neural origin underlying the BOLD signal and provides a key to understanding neural network development through the evolution of complex brain structure.

Other universal properties of the brain topology have also emerged recently with graph analysis. One of them is the small-world feature which maximizes the efficiency of information transferred within a network. This network property has been found in multiple species including humans (Bullmore and Sporns, 2009), NHP (Barttfeld et al., 2015; Garin et al., 2019), rodents (Mechling et al., 2014), and ferrets (Zhou et al., 2016). Moreover, graph-based approaches have clearly revealed a modular nature of human (Sporns and Betzel, 2016), and rodent (Liska et al., 2015) rsfMRI networks, along with evidence of strongly functionally interconnected polymodal areas, exhibiting hub-like properties (Buckner et al., 2009; Liska et al., 2015). Concerning highly connected regions in human, macaque and mouse lemur, the posterior cingulate cortex was found to be critical in these three species with its major functional hubs located in the DMN (Garin et al., 2019). Interestingly, these areas seem to be instead shifted anteriorly in rodents, in which the anterior cingulate and prefrontal areas exhibit robust hub-like properties (Liska et al., 2015; Gozzi and Schwarz, 2016; Garin et al., 2019). This finding is consistent with rodent species lacking an evolutionary homolog of the primate posterior cingulate cortex (Vogt and Paxinos, 2014). Determining the fine-grained topology and contribution of regions critical for network organization and stability across species and evolution could highlight functional patterns that are especially relevant for network stability. Despite the lack of consensus concerning a standardized methodology in mammals fMRI, cross-species studies could provide essential clues toward a better understanding of brain physiology and evolution.

### Animal Preparation and Anesthesia

#### Animal Preparation Impact on Motion and Stress

Functional MRI traditionally relies on temporal changes in hemodynamic parameters, e.g., blood oxygenation level-dependent contrast (BOLD), cerebral blood volume (CBV), or cerebral blood flow (CBF). Functional MRI signals inform on neuronal activity through the evaluation of hemodynamic response i.e., the adaptability of local capillaries to deliver oxygen to active neurons at a greater rate than to inactive neurons. BOLD signal, the most commonly used fMRI parameter, is dependent on the relative levels of oxyhemoglobin and deoxyhemoglobin (oxygenated or deoxygenated blood), which is modulated by local blood volumes and flow. In addition, fMRI acquisitions are highly sensitive to subject movement, specifically at tissue boundaries. In humans, several studies showed that small head motions can produce spurious but spatially structured patterns which drastically impacts RS-FC (Power et al., 2014).

**FIGURE 4:**
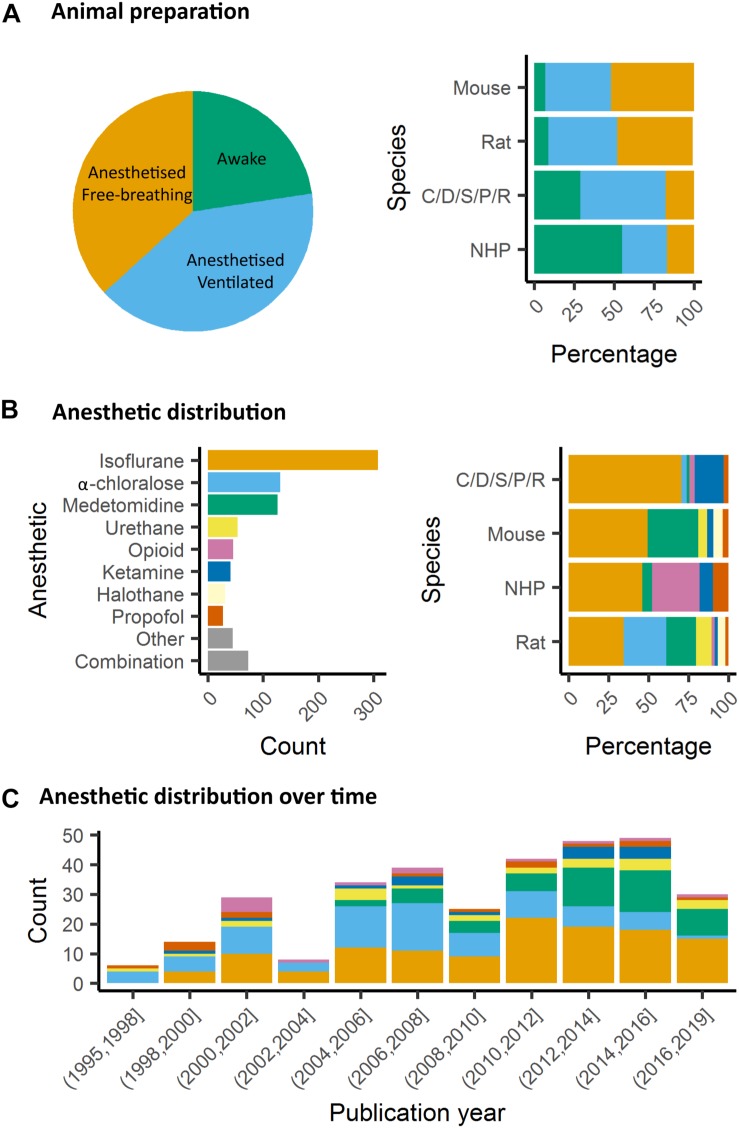
Animal preparation and anesthesia trends. **(A)** Animal fMRI relies mainly on anesthesia to help restrain animals. NHP remain the major species acclimated to awake fMRI. **(B)** Isoflurane is the principal anesthetic used for maintenance during fMRI recordings. However, the distribution of other agents change with species. **(C)** Medetomidine is growing to become the second most used agent behind isoflurane.

In animals, as well, it is critical to control for head motion. As animals are non-compliant species, the most widely used method to control for head stability is to anesthetize the animals and to stabilize the head with bite bar and ear bars (78%, Figure 4A). However, training for awake restraint techniques has been developed in rodents and primates (22%, Figure 4A). These procedures may include acclimation in a scanner environment with an increase of the exposure periods of time. Atraumatic devices such as cylindrical head-holder or flat ear bars can be used to fix the head (Liang et al., 2011). Moreover, head fixes attached to the skull with dental cement provide alternatives that do not require lengthy animal training (Yoshida et al., 2016). In primates, individualized plastic helmets have been constructed based on 3D anatomical images for better stabilization of the head (Belcher et al., 2013). The quality of the mechanical set-up to fix the head is critical: according to Kalthoff et al. (2011), even with carefully fixed heads, motion remains the main source of noise in rat fMRI at 11.75T and it contributes to 30% of the non-neuronal signal variance (60% being attributed to residual noise). This residual motion is related to respiration that represents 5% of the total variance of RS-FC signal (Kalthoff et al., 2011). It can be minimized by artificially ventilating and paralyzing the animal, a process that results in excellent control of the motion artifacts (Ferrari et al., 2012). Beyond motion, either spontaneous or related to ventilation, cardiac motion induces low-frequency BOLD fluctuations and is another source of noise for fMRI signal interpretation (Murphy et al., 2013). In some instances, cardiac responses can eclipse the neuronal response, especially in response to potentially stressful stimuli (Schroeter et al., 2014). Hence decisions to mitigate these strong confounding sources and variations between laboratories remain a major obstacle toward the standardization in animal imaging protocols, decisively more so than in human corresponding experiments.

#### Impact of Anesthesia on Animal Physiology

The global BOLD signal is modulated by heart rate, arterial CO_2_ concentration, and temperature. Different anesthetics modulate various targets in the brain and have different impact on peripheral receptors acting on respiratory or cardiac regulation. Thus, they have different impact on BOLD signal and other hemodynamic readouts. For example, mechanically ventilated rats, for which arterial blood gases (PaCO_2_, PaO_2_) and pH were maintained constant, showed decreased T2^∗^ contrast between veins and parenchyma when anesthetized with isoflurane 2% as compared to medetomidine or ketamine/xylazine. This was explained by increased CBF and vasodilatation in animals under isoflurane (Ciobanu et al., 2012). The use of mechanical ventilation has the advantage of avoiding hypercapnia (increased paCO2) which has an impact on fMRI reproducibility (Biswal et al., 1997; Ramos-Cabrer et al., 2005). Hypercapnia also leads to vasodilation and increased CBF (Xu et al., 2011). The modulation of CBF could explain the decrease of the BOLD response specificity to neuronal activity induced by stimuli (Uhrig et al., 2018). Interestingly, Uhrig et al. showed different impacts of various anesthetics on blood oxygenation in different brain regions. For example, ketamine leads to higher oxygenation in the cortex as compared to the thalamus while the opposite occurs for propofol (Uhrig et al., 2014). This variability may affect the ability to detect networks connecting these regions. The impact of anesthesia on other physiological parameters, such as body temperature and peripheral cardiovascular activity can modulate the quality of the measured functional connectivity. Both these parameters represent strong benefits to be registered and kept stable to assure normal physiological conditions during the acquisition. The body temperature is usually controlled with a heating cradle, pad or any additional heating system, leading to stable reported temperatures. In light of the above, controlling for the temperature, the paCO_2_ and the movement parameters remains essential in assuring the animal stability and the quality of the data. Finally, anesthesia can tightly impact CBF autoregulation in response to peripheral blood pressure changes (Gozzi et al., 2007). Peripheral blood pressure recordings, and the presence of autoregulation, are parameters of critical importance for studies of neuromodulation using drugs, optogenetics and/or chemogenetics-fMRI (e.g., Giorgi et al., 2017), as well as in the case of somatosensory stimulation (Schroeter et al., 2014). This is because transmitter-induced peripherally evoked blood pressure changes, in the absence of physiological CBF autoregulation, can give rise to seemingly regionalized fMRI responses (Gozzi et al., 2007; Reimann H. M. et al., 2018). Future research is required to understand to which extent commonly used anesthetic regimens in rodents do preserve CBF autoregulation. While technically challenging, and invasive, blood pressure recordings can be carried out via femoral arterial cannulation (Ferrari et al., 2015), hence making it possible to understand whether peripheral cardiovascular response and central fMRI activity are temporally correlated.

Several anesthetics are used for animal studies (Figure 4B). They have been classified into several classes according to their targets: GABA_A_ receptors, NMDA receptors, two-pore-domain K+ channels, and other modes of actions. GABA_A_ receptors are the most widely used targets for anesthetics. They are chloride channels that hyperpolarize neurons, making them less excitable and thus inhibiting the possibility of an action potential. Widely used anesthetics as isoflurane, propofol and barbiturates are GABA_A_ receptors agonists (Franks, 2008; Garcia et al., 2010). Each drug within this category displays a subtly unique pharmacological characteristic. For example, isoflurane and sevoflurane have opposite metabolic activities on cerebral blood flow and glucose consumption in various brain regions (Lenz et al., 1998). α-chloralose is widely used in the context of BOLD fMRI because it provides robust metabolic and hemodynamic responses to functional stimulation and is also expected to act on GABA_A_ receptors (Garrett and Gan, 1998). NMDA receptors are other widely used targets. The use of antagonists for these receptors, such as ketamine, is supposed to block excitatory synaptic activity probably leading to anesthesia. This latter may be related to the fact that ketamine binds preferentially to the NMDA receptors on GABAergic interneurons. Ketamine, however, leads to a “dissociative anesthesia” during which the perception of pain is dissociated from the perception of noxious stimuli. Besides, it has psychotomimetic effects at low concentrations, leading to auditory and visual hallucinations (Franks, 2008). Ketamine and other NMDAr antagonists increase regional brain activity and cerebral blood volume, mainly in the anterior cingulate, the thalamus, the putamen, and the frontal cortex (Långsjö et al., 2003; Gozzi et al., 2008; Bonhomme et al., 2012). Two-pore-domain K+ channels are targeted by volatile anesthetics (isoflurane, halothane, nitrous oxide) which have different affinities for subfamilies (TREK-1 or TASK) of these receptors (Patel et al., 1999). These channels modulate the potassium conductance that contributes to the resting membrane potential in neurons. Their opening, therefore, facilitates a hyperpolarizing current, which reduces neuronal excitability and anesthetizes. Among other targets, α2-adrenergic receptor agonists are targeted by xylazine, medetomidine, dexmedetomidine (Sinclair, 2003). The activity of these drugs is related to their action on receptors located in the locus coeruleus and its projections. At this level, they reduce the release of norepinephrine, a neurotransmitter that is necessary for arousal. The anesthesia induced by these compounds resembles the state of non-REM sleep, i.e., the first four of the five stages of the sleep cycle (Franks, 2008).

#### Impact of Anesthetics on Neuronal Network Organization in Rodents

In rodents, isoflurane and medetomidine are currently the most commonly used anesthetics (Figures 4B,C). Importantly, isoflurane is almost systematically used for anesthesia induction, specifically in rodents. Variations in the induction time may lead to a lasting effect on brain function, even though anesthesia is switched to another agent (Magnuson et al., 2014). In addition to their different mechanisms of action (GABA_A_ receptors agonist for isoflurane and α2 adrenergic receptor agonists for medetomidine), they have opposite vaso-properties (vasodilatation for isoflurane and vasoconstriction for medetomidine) which could impact neurovascular coupling differently. In rodents, isoflurane seems to preserve the interhemispheric and cortico-cortical FC but only at low doses (∼1%) (Wang et al., 2011; Grandjean et al., 2014; Uhrig et al., 2014; Bukhari et al., 2017). Medium to high doses induce burst-suppression effects which are reflected in an increase in the global signal (Liu et al., 2011, 2013; Grandjean et al., 2014). Medetomidine seems to present opposite effects such as a cortico-cortical disruption and a pronounced striatal FC (Grandjean et al., 2014; Bukhari et al., 2017; Paasonen et al., 2018). The effect of isoflurane and medetomidine and other anesthetics on the thalamo-cortical FC is still debated. Several studies suggested that a combination of isoflurane and medetomidine (med/iso) at low doses is the best compromise (Table 1, med/iso) to preserve FC and to recapitulate network properties of the awake state (Grandjean et al., 2014). However, this combination appears to inhibit thalamo-frontal cortical connectivity, when compared to connectomic estimates of the mouse connectome (Grandjean et al., 2017b). A number of studies in control and transgenic mouse models have been carried out with low doses of halothane (Sforazzini et al., 2014; Liska et al., 2015, 2018; Bertero et al., 2018; Gutierrez-Barragan et al., 2018; Pagani et al., 2019). This inhalational anesthetic produces stable and long-lasting RS-FC correlation recapitulating patterns of connectivity observed with med/iso combination (Grandjean et al., 2017b), with the advantage of robustly preserving thalamo-frontal connectivity, an effect that makes it especially apt for the investigation of prefrontal circuitry and the rodent default mode network (Bertero et al., 2018). However, the hepatotoxic properties of this compound have led its banning in most countries, preventing widespread use of this anesthetic regimen. Other anesthetics used in rodents (propofol, urethane, α-chloralose) are presented in Table 1. They are not further discussed here as they showed ambiguous effects on RS-FC and are no longer recommended. Notably, RSNs in mice were shown to converge in a multi-center comparison (Figure 1; Grandjean et al., 2019a), irrespective of anesthesia regimen, indicating to some extent that network properties are retained between different conditions.

**TABLE 1:** Anesthetics effects on the functional connectivity in rodents.

**Anesthetics**	**Doses**	**Comparison**	**Effects**	**Studies**	**Species**
Isoflurane	1%	vs. the awake state	Preserve interhemispheric FC	Jonckers et al. (2014)	Mice
		vs. anesthetics	Cortical and thalamo-cortical FC preserved but disruption of striatal FC	Grandjean et al. (2014)	
			Cortico-cortical FC preserved but disruption of thalamo-cortical FC	Bukhari et al. (2017)	
	1–2%	Increasing doses	Disruption of interhemispheric FC with increasing doses	Bukhari et al. (2018)	
	1.3%	vs. the awake state	Cortico-cortical and striatal FC increase	Paasonen et al. (2018)	Rats
Medetomidine	0.1 mg/kg	vs. anesthetics	Disruption of thalamo-cortical FC but pronounced striatal FC	Grandjean et al. (2014)	Mice
			Thalamo-cortical FC preserved but disruption cortico-cortical FC	Bukhari et al. (2017)	Mice
		vs. the awake state	Cortico-cortical FC decreased	Paasonen et al. (2018)	Rats
Med/iso	0.05 mg/kg; 0.5%	vs. anesthetics	Preserved FC	Grandjean et al. (2014)	Mice
				Bukhari et al. (2017)	
	0.06 mg/kg; 0.5%	vs. the awake state	Thalamo-cortical and intra-subcortical FC decrease	Paasonen et al. (2018)	Rats
Urethane	2.5 g/kg	vs. the awake state	Disruption of interhemispheric FC	Jonckers et al. (2014)	Mice
	1.5 g/kg	vs. anesthetics	Cortical and thalamo-cortical FC preserved but disruption of striatal FC	Grandjean et al. (2014)	
	1.25 g/kg	vs. the awake state	Replication of the awake state	Paasonen et al. (2018)	Rats
α-chloralose	120 mg/kg	vs. the awake state	Disruption of interhemispheric FC	Jonckers et al. (2014)	Mice
	60 mg/kg	vs. the awake state	Cortico-cortical FC suppression	Paasonen et al. (2018)	Rats

*Review of five studies between 2014 and 2018.*

#### Impact of Anesthetics on Neuronal Network Organization in Primates

In primates, isoflurane is the most used anesthetic (Vincent et al., 2007; Hutchison et al., 2013b; Miranda-Dominguez et al., 2014; Grayson et al., 2016). As in rodents, lower dose and shorter anesthesia duration are associated with an increased ability to detect RS-FC (Table 2; Barttfeld et al., 2015; Uhrig et al., 2018). Also, one should keep in mind that a direct comparison of the impact of anesthetics on cerebral networks is difficult because anesthesia depth also modulates networks and can lead to misinterpretation of the results.

**TABLE 2:** Anesthetics effects on the functional connectivity in primates.

**Anesthetics**	**Doses**	**Comparison**	**Effects**	**Studies**	**Species**
Isoflurane	1–2.75%	Increasing doses	Disruption of interhemispheric FC after 1.5%	Hutchison et al. (2014)	*Macaca fascicularis*
	0.89–1.19%	Duration effect	Reduction of the DMN FC with a prolonged administration	Li and Zhang (2018)	*Macaca mulatta*
Ketamine	20 mg/kg	vs. the awake state	Preservation of positive FC but average positive FC reduced	Uhrig et al. (2018)	*Macaca mulatta*
Sevoflurane	2.2–4.4 vol%	vs. the awake state	Average positive FC reduced	Uhrig et al. (2018)	*Macaca mulatta*

*Review of five studies between 2014 and 2018.*

### Data Acquisition

**FIGURE 5:**
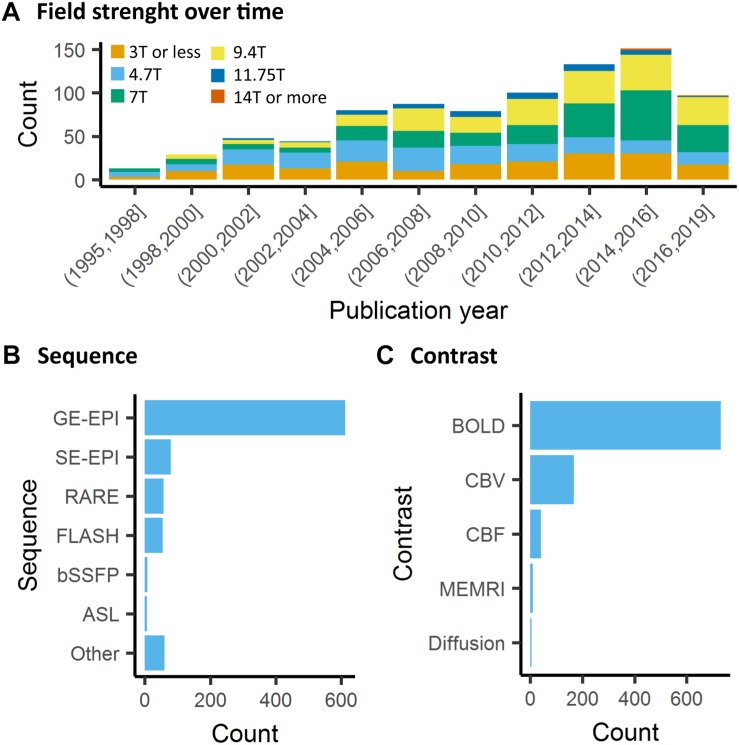
Data acquisition. **(A)** There is a general trend toward higher strength of the main magnetic field in animal fMRI over time. In the past decade, the majority of studies were performed on 7T and 9.4T systems. **(B)** The acquisitions relied mainly on gradient echo EPI for the acquisition, while older studies either used FLASH or RARE sequences. **(C)** BOLD is the most commonly used contrast in animal studies. The availability of iron nanoparticles in animal studies explains the relative high incidence of CBV contrasts.

Contrary to human fMRI, which is carried mostly at 1.5T, 3T, and in rarer cases at 7T, animal fMRI is carried at a variety of field strengths, with 7T and 9.4T being the most frequently encountered field strength (26 and 25% respectively, Figure 5A). The availability of ultra-high field strength small-bore systems in rodents further increase this range, with fMRI being recorded as high as 15.2T (Jung et al., 2019). While fewer animal MRI system vendors exist compared to human systems, this apparent similarity is compounded with a greater range of coil designs, including home-made coils or cryogenic coils (Baltes et al., 2011), which provide an additional source of variation among the animal studies. Whilst these factors are determined by the center where the acquisitions are performed, even greater variability comes in in the form of sequence parameters and the resulting contrasts across the different studies. This is exemplified in a report by Grandjean et al. which indicated cortical signal-to-noise ratios ranging from 20 to 400 in mice fMRI acquired at different centers (Grandjean et al., 2019a).

Neuronal activity induces vasodilation in surrounding capillaries and arterioles, which may propagate further up- and downstream toward arteries and draining veins. The resulting increase in CBF and CBV and blood oxygenation forms the basis of imaging strategies for fMRI. The most commonly used fMRI method is based on the BOLD contrast (Ogawa et al., 1990). BOLD contrast results from the paramagnetic properties of deoxyhemoglobin, which causes magnetic susceptibility effects inside blood vessels as well as in their surrounding tissue that can be detected with T2- or T2^∗^-weighted sequences (Norris, 2006; Kim and Ogawa, 2012). Deoxyhemoglobin concentration increases dramatically from the arterial (<5%) to the venous side (∼40%) of the vascular tree due to the extraction of oxygen in the capillaries (Vovenko and Sokolova, 1998), which makes BOLD imaging particularly sensitive to capillaries, venules and veins. In healthy brain tissue, the neuronal activity typically induces an increase in CBF with resultant increased oxygen delivery that exceeds the decrease in oxygen due to capillary oxygen extraction. As a result, deoxyhemoglobin concentration in the capillaries and veins decreases, giving rise to a positive BOLD response in T2- or T2^∗^-weighted images.

The most frequently used BOLD-weighted fMRI sequence in rodents is T2^∗^-weighted gradient echo (GE) echo planar imaging (EPI) (Figure 5B). GE-EPI provides a relatively high contrast-to-noise ratio (CNR), which increases with field strength. At field strengths ≥ 7T, the intravascular contribution to the GE BOLD signal is negligible and signal changes scale almost linearly with echo time (TE) (Yacoub et al., 2003; Han et al., 2019). For optimal BOLD CNR, TE is typically set equal to the average gray matter tissue T2^∗^ value (for an overview of brain tissue T2 and T2^∗^ values we refer to Uludağ et al. (2009) and Han et al. (2019). The disadvantage of using GE-EPI for rodent fMRI, however, is its sensitivity to susceptibility artifacts, which are most prominent near air cavities such as the ear canals and around the olfactory bulb, particularly at long TE and high field strength. Furthermore, GE-EPI is highly sensitive to large veins (Uludağ et al., 2009), which makes this sequence spatially non-specific as neurovascular coupling occurs at the level of the capillaries. This has been clearly demonstrated by fMRI studies in rats subjected to electrical stimulation of the forepaws, where the highest GE-EPI BOLD response is observed in the outer layer of the somatosensory cortex where pial vessels are located (Mandeville and Marota, 1999; Han et al., 2019), while neuronal activation mostly occurs in deeper cortical layers. The relative contribution of capillaries to the BOLD signal increases with field strength but remains dominated by the macrovasculature even at 15.2T (Han et al., 2019).

Spatial specificity for neuronal activity can be increased by using spin-echo (SE) EPI for BOLD fMRI (Norris, 2012; Han et al., 2019). SE BOLD is particularly sensitive to small vessels, as signal around large vessels is largely refocused by the 180° pulse. The relative contribution of the microvasculature increases with field strength and TE, and may be further increased by introducing diffusion gradients that reduce the remaining intravascular contribution from large vessels (Kim and Ogawa, 2012). To maintain spatial specificity, EPI train length should be reduced to a minimum to avoid introducing T2^∗^ effects (Goense and Logothetis, 2006). In the absence of intravascular contributions to the SE BOLD signal, CNR increases almost linearly with TE, achieving the best contrast when TE equals gray matter tissue T2. SE-EPI images show reduced sensitivity to susceptibility artifacts compared to GE-EPI. However, SE-EPI also comes with lower BOLD CNR, and longer acquisition times.

Since BOLD contrast depends on the TE of the sequence, multi-echo GE sequences have been proposed for BOLD fMRI data acquisition. In multi-echo EPI (ME-EPI), one excitation pulse is followed by acquisition at multiple TEs (Speck and Hennig, 1998). Short TE results in high signal intensity, minimal signal dropout but low CNR, whereas longer TE results in lower signal intensities, more signal dropout but higher CNR. The multi-echo approach has two main applications. First, images acquired at different TE can be combined to optimize the BOLD contrast per region (Posse et al., 1999; Poser et al., 2006), since T2^∗^ varies across the brain (Hagberg et al., 2002; Peters et al., 2007). Second, identifying TE-dependent and TE-independent signals can help to separate BOLD T2^∗^ signal fluctuations and noise (Kundu et al., 2012). The shortened T2^∗^ at high field strength, often used for preclinical imaging, provides less time for image acquisition at additional TEs and limits the time between adjacent TEs. Still, ME-EPI at three different TEs without acceleration is feasible for fMRI in rodents at 9.4T and 11.7T, with a TR of 1.5–3 s and acceptable spatial resolution (Kundu et al., 2014; Grandjean et al., 2017a, b).

Beside EPI, the balanced steady-state free precession (bSSFP) sequence enables acquiring BOLD-like contrast images at short TE (=TR/2), making these images relatively insensitive to signal dropouts and artifacts often seen in GE-EPI. The origin of the bSSFP contrast is, however, complex since it does not only depend on T1 and T2 but also on the flip angle, repetition time and off-resonance values (Miller, 2012). Functional MRI using bSSFP sequences can be performed in the so-called transition-band or the pass-band (Miller, 2012). Functional transition-band bSSFP is sensitive to alterations in voxel off-resonances induced by changes in deoxyhemoglobin concentration. At short TE it provides T2-weighted contrast (Scheffler et al., 2001), whereas at long TE the image contrast is mainly T2^∗^-weighted (Miller et al., 2007). Larger signal increases in response to neuronal activation have been measured compared to GE-EPI (Scheffler et al., 2001; Miller et al., 2006). However, transition-band SSFP is also sensitive to field inhomogeneities due to its sensitivity to off-resonances, making whole-brain coverage from anterior to posterior difficult to achieve (Miller, 2012). Furthermore, it is sensitive to physiological and time-varying noise (Miller, 2012). Pass-band bSSFP has been more widely used for fMRI (Miller and Jezzard, 2008; Scheffler and Ehses, 2016). Similar to transition-band bSSFP, its contrast origin shifts from BOLD T2 effects at short TE to BOLD T2^∗^ effects at long TE. However, the pass-band SSFP sequence is less sensitive to field inhomogeneities, and sensitivity to physiological noise can be lower than with GE-EPI (Miller et al., 2007). At short TE, an additional advantage is the suppression of BOLD sensitivity in large draining veins, making the sequence more selective to microvasculature contribution compared to GE-EPI (Báez-Yánez et al., 2017). However, bSSFP sequences have lower BOLD CNR than conventional GE-EPI at short TE (Miller et al., 2007; Zhong et al., 2007), and at long TE, banding-artifacts appear due to field inhomogeneities and macrovascular contributions increase. Consequently, the use of this sequence has so far remained marginal (Figure 5B).

Although BOLD contrast is mostly used for fMRI, alternative methods that directly measure CBV or CBF, are available (Figure 5C). CBV can be measured with the use of exogenous iron oxide-based contrast agents (Mandeville et al., 1998; Chen et al., 2001). Iron oxide nanoparticles used for CBV contrast exhibit strong r2 and r2^∗^ relaxivity, do not cross the intact blood-brain barrier, and have a long blood circulation half-life in the order of hours (Chen et al., 2001). Intravenous administration of nanoparticulate iron oxide introduces magnetic susceptibility effects within the vasculature and its surrounding tissue, which, at sufficiently high dose, are much larger than the effects of deoxyhemoglobin. As a result, intravascular T2^∗^-weighted signal becomes negligible, while the extravascular T2^∗^-weighted signal becomes highly sensitive to changes in CBV (Mandeville, 2012). An increase in CBV, as induced by neuronal activation, increases magnetic susceptibility within the imaging voxel, giving rise to negative CBV-dependent contrast in T2^∗^-weighted images. CBV-dependent contrast is independent of field strength and most optimal when iron oxide injection causes a drop of 40–60% in signal intensity with respect to baseline (Mandeville, 2012). Since baseline signal intensity is strongly dependent on TE, contrast dose should be adjusted to the TE as well. A relatively high dose of contrast agent allows the use of short TE with the advantage of a reduction in susceptibility artifacts (Mandeville et al., 2004). The most commonly used imaging sequence for CBV contrast is GE-EPI, which, in contrast to BOLD GE-EPI, is particularly sensitive to changes in small vessels (arterioles, capillaries and venules). This, which is due to the strong magnetic susceptibility effects of the iron oxide, causes the extravascular signal from tissue surrounding large vessels to be largely eliminated. CBV-weighted fMRI is therefore considered more spatially specific to neuronal activity than GE BOLD fMRI. This has been clearly demonstrated in rats subjected to electrical forepaw, in which a spatial shift in the maximum contrast-to-noise ratio was observed from the cortical surface with GE BOLD fMRI to deeper layers of the somatosensory cortex with GE CBV-weighted fMRI (Mandeville and Marota, 1999; Keilholz et al., 2006). SE-EPI is typically not used for CBV-weighted fMRI as CNR is lower than with GE-EPI, and CBV changes in small vessels are underestimated (Mandeville et al., 2007).

Cerebral blood flow can be measured non-invasively with Arterial Spin Labelling (ASL), which uses radiofrequency pulse(s) to magnetically label the blood water in major arteries by inverting the longitudinal magnetization (Williams et al., 1992). After a waiting period, the labeled blood water exchanges with brain tissue water, leading to T1 shortening in the imaging plane. Subtracting a second scan without labeling results in an image with only the signal from the labeled inflowing spins. There are two main types of ASL: continuous ASL (cASL) and pulsed ASL (pASL) (Borogovac and Asllani, 2012). cASL sequences include a long labeling pulse and provide high signal-to-noise ratio but low labeling efficiency. In comparison, pASL involves short inversion pulses with high labeling efficiency but low signal-to-noise ratio. A practical advantage of pASL is that short inversion pulses are more easily implemented in ASL protocols. To combine the higher labeling efficiency of pASL and higher sensitivity of cASL, pseudo-continuous ASL (pCASL) was developed (Silva and Kim, 1999; Wu et al., 2007; Dai et al., 2008; Borogovac and Asllani, 2012), and further optimized with multi-phase image acquisitions to tackle rodent-related difficulties with variations in labeling efficiency across different vessels to prevent erroneous calculation of CBF (Larkin et al., 2018). Since EPI is the main read-out for ASL, the presence of a BOLD effect should be taken into account in ASL-based fMRI studies (Lu et al., 2006). Compared to BOLD fMRI, ASL-based fMRI provides about one-third of the contrast-to-noise ratio (Lu et al., 2003), has low temporal resolution and is more challenging to implement (Detre and Wang, 2002). On the other hand, ASL provides stable noise levels – enabling measurement of slow variations in brain function (Aguirre et al., 2002; Wang et al., 2003) – shows less intersubject variability (Tjandra et al., 2005), and is more sensitive to arterioles and microvasculature than to large draining veins (Silva et al., 1999; Tjandra et al., 2005).

By far the majority of rodent fMRI studies are executed with one of the abovementioned fMRI approaches that are based on the hemodynamic response to neuronal activation. Alternative fMRI methods aimed at more specific detection of neuronal responses have been developed, such as manganese-enhanced fMRI (Lin and Koretsky, 1997) and diffusion-weighted fMRI (Tsurugizawa et al., 2013) but these approaches have been hampered by non-uniform or limited sensitivity, low temporal resolution and uncertainties about the underlying mechanisms (Rudrapatna et al., 2012; Silva, 2012). Correspondingly, the application of these methods has so far remained marginal (Figure 5C). Recent developments in diffusion-weighted fMRI in rodents are likely to give rise to a renewed interest in the method (Abe et al., 2017; Nunes et al., 2019).

### Data Analysis

#### Pre-processing

**FIGURE 6:**
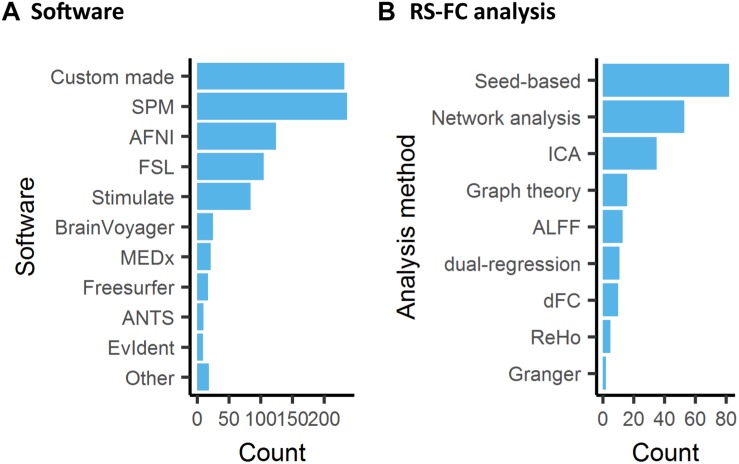
Software used for data analysis of animal fMRI and functional connectivity analysis. **(A)** Custom made software or combination of existing software pipelines remained the most common approach to animal fMRI data processing, while SPM was the most used software package used. **(B)** Resting-state fMRI in animals is mainly analyzed with seed-based analysis.

Image pre- and post-processing are an integral part of every fMRI study. Pre-processing refers to a number of steps to correct for artifacts and normalize data, e.g., motion correction, temporal filtering and co-registration to a reference template. A number of dedicated software packages are designed, usually for human studies, to carry out these functions. With differences in the precision and performance of the various tools available, e.g., motion correction (Morgan et al., 2007) or registration (Klein et al., 2009), the user selection of tools within data analysis is a non-negligible source of bias and variability between studies. Interestingly, an analysis in human fMRI revealed that 223 unique analysis pipelines were used to process data in 241 studies, implying that nearly every study is carried out with an individualized pipeline (Carp, 2012). Efforts to develop unified open-source pre-processing pipelines for human fMRI, e.g., fMRIPrep (Esteban et al., 2019), have yet to reach widespread adoption. In animals, we observed that a large number of studies relied on custom made pre-processing functions (26%, Figure 6A). SPM was the most common software package used for the analysis (27%). The preponderance of custom made software, as well as the combination of functions from several software suits in animal fMRI research, may be explained by the fact that specific functions were designed for the human brain. The pervasive use of *ad hoc* pipelines, encouraged by the lack of dedicated animal pipelines, is a major obstacle to results comparisons between centers.

#### Templates and Atlas Selection

Registration of fMRI results to a common reference space is an integral part of the pre-processing and enables unbiased group-level statistical analysis at a voxel-wise level. In human neuroimaging, standard space and coordinate systems are routinely used to report results in both figures and tables. In animals, we found that the vast majority of the studies did not register fMRI data to standard space (64%) while 24% relied on *ad hoc* templates. While this step ensures optimal registration due to similar image contrast, resolution, and orientation, this adds extra challenges in comparing across studies. Contrary to the ubiquitous Montreal Neurological Institute reference space in human (Mazziotta et al., 2001), animal templates have failed to reach a consensus yet, despite efforts to implement standards such as the Waxholm space (Johnson et al., 2010). This is exemplified by the various templates used in animal studies. In rats, five studies relied on (Schweinhardt et al., 2003), nine used (Schwarz et al., 2006), five used (Valdés-Hernández et al., 2011), and two used (Nie et al., 2013). In NHP, ten studies were normalized to Van Essen et al. (2012), ten used (Saleem and Logothetis, 2012), six used (McLaren et al., 2009), and six normalized to Rohlfing et al. (2012). In mice, seven studies were normalized to Janke and Ullmann (2015), four studies used (Lein et al., 2007), and two used (Dorr et al., 2008). More importantly, none of the studies reported three-dimensional coordinates for clusters or slice positions, rendering the precise comparison between studies impractical. Registration between rodent or NHP brains is, however, a computationally much easier challenge than between human brains due to the simpler and less idiosyncratic cortical folding (NHP) or lissencephalic cortex (rodents). The choice of atlases and the level of parcellations also have large implications for network analysis and graph theory metrics (see below).

#### Regional and Network Level Analysis of Resting-State fMRI

Stimulus-evoked, pharmacological, DBS, and opto-/chemogenetics fMRI studies are almost systematically analyzed with voxel-wise statistics where the time series at every voxel is assessed with an independent model, usually a model of the hemodynamic response to the stimulus/injection paradigms. This is often complemented with ROI analysis of the evoked response. In comparison, RS-FC is paradigm free, hence often relies on intrinsic models to infer connectivity or associated metrics. Consequently, there are several analysis methods that have been developed primarily in the human literature but which can easily be applied to animals as well (Figure 6B). Approaches range from hypothesis-driven (e.g., seed-based analysis) to data-driven (e.g., Independent Component Analysis, ICA) and can be applied at the level of networks or of particular ROI. Some metrics describe the relationship between areas; others are based on features of the low-frequency BOLD fluctuations from a single region. Here we provide a brief overview of some prominent methods and reflect on their interpretations.

#### Seed-Based Correlation

Seed-based correlation is the most intuitive of the analytical methods and the most commonly used in animals (Figure 6B). A seed region can be defined based on function or anatomy and range in size from a few voxels to a parcel from a brain atlas. The time courses from each voxel in the seed are averaged together, and then the correlation is calculated between the averaged seed time course and the time course from every other voxel in the brain. The resulting correlation values can be displayed at the location of each voxel, producing a correlation map (Pawela et al., 2008; Williams et al., 2010; Kalthoff et al., 2011; Liang et al., 2013; Sforazzini et al., 2014; Zerbi et al., 2015; Paasonen et al., 2018). Average values can then be measured for ROIs. Alternatively, the signal from the desired target area can be measured and correlated with the seed time course to directly examine the connection from a particular pair of areas. Seed-based correlation is a low-level metric and thus relatively easy to interpret and to assess for quality. Reference maps for several seeds in the mouse brain are provided in Grandjean et al. (2019a). As with any measurement, it can be affected by the relative levels of signal and noise. While correlation is robust to differences in amplitude in the two signals, a reduction in BOLD amplitude can go hand in hand with an increase in non-neural noise, which does affect correlation values (Keilholz et al., 2016).

#### Independent Component Analysis

Independent Component Analysis is a data-driven way to identify networks within the brain. It is widely used in the human neuroimaging community and does not rely on the definition of a seed. Instead, it identifies a number of statistically independent networks that can be mapped spatially to the brain (Hutchison et al., 2010; Lu et al., 2012; Jonckers et al., 2014; Sforazzini et al., 2014). One of the challenges of the technique is that it is not immediately apparent how many components should be used. As more components are included, the resulting networks fragment into separate areas, and it may sometimes be necessary to combine components to recompose the full network structure. Accordingly, distributed networks of the rodent brain that are robustly identified using seed-based mapping, such as the DMN (Sforazzini et al., 2014), are only detectable with low-dimensional ICA, and are typically segregated into functional sub-portions when a more canonical number of components is selected. As such, the choice of component number is one of the sources of variability across experiments, but it is at least somewhat mitigated by the observation that the same networks can typically be detected in most studies, despite the occasional fragmentation. Other choices that contribute to variability across studies include whether ICA is performed on the entire group of animals at once, or on subgroups (e.g., mutant vs. wild type mice). If performed on the whole group, a single set of components is obtained and its strength can be examined in each group. One risk of this approach is that the component structure might be driven by one of the subgroups. If ICA is performed on subgroups, multiple sets of components are obtained with different spatial extents and strengths, making comparisons more difficult. ICA provides spatial maps of components and can be considered a mid-level parameter. Additional analysis is needed to examine the strength of the connectivity within or between networks obtained from ICA and is often calculated using correlation. Strict criterion for the identification should be encouraged, such as those proposed in Zerbi et al. (2015), to promote comparable classifications between studies.

#### Amplitude of Low Frequency Fluctuations

The amplitude of low-frequency fluctuations (ALFF; Zang et al., 2007) and fractional ALFF (Zou et al., 2008) represent the amplitude of the BOLD fluctuations within specific frequency bands, e.g., the 0.01–0.08 Hz range. For fALFF, the amplitude of the fluctuations in the range of interest is normalized by the amplitude of the full frequency range. Both of these measures give an estimate of the strength of the BOLD fluctuations used to map RS-FC, and may include both neural contributions and vascular effects like cerebrovascular reactivity (Golestani et al., 2016). ALFF and fALFF are low-level parameters. In rodents, they are most commonly used to compare across experimental groups (Yao et al., 2012; Chang et al., 2018; Wen et al., 2018).

#### Regional Homogeneity

Regional Homogeneity (ReHo) is a measure of the local correlation between adjacent voxels (Zang et al., 2004). Similarly to ALFF, and contrary to the majority of the other methods described here, ReHo is a measure that informs on the local signal coherence strength, but not of distal connectivity. The method has been effectively applied in rodents (Wu et al., 2017; Li et al., 2018) and NHP (Rao et al., 2017), such as to describe anesthesia effects on the mouse brain (Wu et al., 2017). ReHo is also a low-level parameter and is relatively simple to interpret.

#### Whole Brain Analysis

When pursuing a whole brain analysis of RS-FC data, the first question to be answered is that of parcelation. In theory, an analysis could also be performed using each voxel as an independent region, but the signal is noisy and spatially redundant. It is generally agreed upon to group voxels in some way to reduce the dimensionality of the ensuing analysis. The choice of the atlas is often dictated by the level of detail achieved. Parcelation by atlas is an anatomical approach, even though the atlas may be derived from functional information. Another possibility is to perform a functional parcelation, either by clustering or by using a dimensionality reduction algorithm like ICA (Jonckers et al., 2014; Medda et al., 2016). These approaches incorporate information carried by the resting BOLD signal instead of relying on spatial alignment. Following parcelation, other analysis methods are typically applied. One common approach is to calculate the correlation between the average time courses of every possible pairs of ROI. This is similar to seed-based correlation except that the regions of the atlas are used as seeds and targets. Partial volume effects can, therefore, distort the results. The correlation values for the whole brain are often displayed as matrices, where each line shows the correlation for a given ROI with all other possible ROIs. It is then possible to test correlation matrices for differences across groups (although correction for multiple comparisons is essential) or to calculate additional summary metrics using graph theory approaches, described in the next section.

#### Graph Theory

The brain can be viewed as a network, with ROI serving as nodes that are connected by edges whose weight is determined by a measure of RS-FC, usually correlation. From this perspective, an entire arsenal of graph-theoretical metrics can be used to describe the network of the brain. These range from mid-level metrics such as degree (the number of edges that connect to a node) to high-level metrics such as modularity that describe the community structure of the brain. For an overview, see Bullmore and Sporns (2009). High-level metrics provide a convenient summary of the large-scale functional architecture of the rodent functional connectome, amenable to cross-species translation (Stafford et al., 2014; Liska et al., 2015; Bertero et al., 2018). They, however, can be influenced by low-level parameters, such as global correlations, and arbitrary parameters such as matrix sparsity whose effects cascade through the analysis and complicate interpretation.

#### Non-stationary Analysis

In recent years, interest has grown in examining fMRI data beyond the stationary assumptions made by several of the methods described above, also referred to as dynamic functional connectivity. The simplest approach is to use a windowed version of the image time series to calculate the metrics described above (e.g., correlation) (Keilholz et al., 2013). The window is moved along the time series and the calculation is repeated at different time points. A number of studies have examined the effects of window size, shape, and step size, but the ideal parameters remain difficult to pin down (Hindriks et al., 2015; Leonardi and Van De Ville, 2015; Shakil et al., 2016, 2018; Grandjean et al., 2017a). Windows can be applied to the time courses from a particular ROI or from the whole brain, where they are often summarized as a series of matrices (Allen et al., 2014). Other methods can be used to look at the co-occurrence of the individual events that drive RS-FC (Petridou et al., 2013; Liu et al., 2018) or at large-scale repeated patterns of activity (Majeed et al., 2011; Grandjean et al., 2017a; Belloy et al., 2018), offering the possibility of mapping RS-FC non-correlative terms. There are major methodological considerations to such analysis (Laumann et al., 2017; Liégeois et al., 2017). Yet, some of the crucial confounds, specifically head motion, are less applicable to animal studies in anesthetized or paralyzed animals. It emerges that non-stationary patterns are reproducible in both human and rodent datasets (Abrol et al., 2017; Grandjean et al., 2017a; Gutierrez-Barragan et al., 2018). These represent promising emerging methods to investigate the RS-FC signal beyond the stationary hypothesis which characterizes the methods discussed above.

#### Functional Connectivity Metrics and Interpretation

Choices of anesthesia and pre-processing pipeline have the greatest effect on the ability to compare results from different studies (Pan et al., 2015). However, the wide variety of analysis methods available also plays a role in our interpretation of the results. While the choice of analysis is ultimately dictated by the question of interest, there are steps that can be taken to promote standardization across studies. For example, reporting baseline metrics like correlation along with higher-level metrics like modularity would assist with interpretation and comparison to other studies. In human neuroimaging, a test–retest examination of varying RS-FC methods has highlighted reliable methods (Zuo and Xing, 2014), including dual-regression (Filippini et al., 2009). There are a few explicit examinations of test–retest reproducibility in rodents that undergo the same experimental protocols, providing insight into the level of reproducibility that might be expected. Zerbi et al. (2015) found an *R*^2^ value of ∼ 0.7 for optimally processed data from mice under medetomidine/isoflurane combination (Zerbi et al., 2015). Kalthoff et al. (2013) showed that the spatial localization of ICA components shares a common core, particularly under medetomidine sedation. Converging ICA and seed-based maps derived from multiple-datasets are available in the mouse as quality assurance references (Grandjean et al., 2019a). Nevertheless, substantial variability in the correlation coefficients from different studies is present, depending on pre-processing steps, ROI definition, and other factors.

### Statistics and Resource Sharing

The statistical analysis carried out by the neuroimaging community has been under increasing scrutiny following reports of inflated false-positive rates in the parametric statistical models traditionally used (Eklund et al., 2016). To assess the emergence of non-parametric voxel-wise statistics, we recorded the occurrence of non-parametric statistics. We found only 12 mentions of such tests out of 868 studies. This low incidence is indicative of comparable trends in the corresponding human neuroimaging field. Differences between studies are accentuated as voxel-wise statistics in animal studies have been corrected with varying degrees of stringency, such as correcting by arbitrary *ad hoc* cluster size or *p*-value threshold. These render the comparison between studies opaque. To overcome these limitations and to permit meta-analysis, NeuroVault (Gorgolewski et al., 2015) offers a resource to publish statistical maps prior to statistical thresholds, leaving the users to explore, reinterpret, and repurpose these results. Unfortunately, such resources are not yet available to animal neuroimaging studies. The advent of RS-FC and network analysis is another source of dissension in the statistical analysis. With fine-grain ROI sets, the number of edges increases dramatically, hence the number of univariate tests and the need to correct for multiple comparisons. There, no consensus currently exists to effectively account for multiple comparisons and the heightened level of false positives ensuing.

The growth of the human neuroimaging community has been fueled by large datasets made publicly available in online repositories (Nichols et al., 2017). Making raw data available is becoming a requirement by the funding bodies, academic center, and the journals. In spite of these requirements, we only found 15 mentions of data availability, among 868 studies, seven of which upon reasonable request to the senior author. Publication of datasets on established repositories ensures lasting availability of the dataset and unbiased distribution. Databases such as XNAT^2^ (Herrick et al., 2016) and Openneuro^3^ (Poldrack et al., 2013) are becoming increasingly user-friendly, including standardized formats that allow for the easy organization and retrieval of functional and anatomical data (Gorgolewski et al., 2016). Importantly, potential reticence in human neuroimaging to share material to protect subject privacy do not apply in animal research. Importantly, shared material allows for in-depth scrutiny of published results and hence strengthen the trust in the published results and facilitate meta-analysis.

## Conclusion and Outlook

With this study, we describe the general trends in animal functional neuroimaging and reflect on emerging collective efforts driving toward larger multi-center studies and a desire for the adoption of standards and good practices in the field. Several issues highlighted above are specific to the animal imaging field, such as those related to opto-/chemo-fMRI study designs, anesthesia, and data preprocessing. Others are shared with the human neuroimaging field, including acquisition sequences and data analysis methods, but still contain specific considerations for the animal imaging community. A general consensus on several acquisition procedures within the community is unlikely to be reached, especially on contentious topics such as anesthesia and animal preparation. Nonetheless, we report general trends which indicate some degrees of consensus. For instance, isoflurane and medetomidine and/or their combination represent an increasing proportion of the studies performed in anesthetized rodents, supported with increasing evidence from the literature. Sequences and contrasts are also reaching consensus, as the overwhelming majority of the studies were acquired using GE-EPI and BOLD contrast, predominantly at high fields such as 7T and 9.4T. Importantly, a number of aspects emerge which are currently lacking within our community and which could easily be implemented to greatly ameliorate how results are interpreted. While modest, these first steps will be necessary for the adoption of standards, replication studies, and meta-analysis.

Firstly, the systematic sharing of raw datasets upon publication is the easiest milestone to be achieved within our community. It is often requested by both funding agencies and publishers alike. Yet, less than 1% of the studies were published with its raw data. This represents a severe loss to our community as it prevents the repurposing of material and the critical re-assessment of past results. Arguably, a number of debates regarding methodological aspects of fMRI acquisitions would find a rapid resolution if the material were accessible by the community for in-depth scrutiny. Moreover, a number of variations in data processing highlighted above would be rendered moot as the material could be re-analyzed with other pipelines to confirm or compare results.

The second aspect within acceptable reach in the animal neuroimaging community is the adoption of common references spaces and the reporting of accurate coordinates in both figures and tables, as is common practice in human studies. Despite several templates being available for mice, rats, and NHP (Bakker et al., 2015), no consensus has yet emerged. The reliance on *ad hoc* templates further hinders the adoption of standard templates. While Paxinos and Franklin mice (Paxinos and Franklin, 2012) and Paxinos and Watson rats atlases (Paxinos and Watson, 1982) are systematically referred to, activation clusters have not been reported with respect to their three-dimensional coordinates reported in these atlases. Hence, the adoption of exact three-dimensional coordinate systems, together with tools to convert from one system to another would greatly ameliorate how results in animal neuroimaging studies are reported, and would also among other enable meta-analyses. This should also be accompanied with easily accessible, fully validated open-source data processing toolboxes tailored for animal fMRI data, similarly to what is available in human neuroimaging (Esteban et al., 2019).

Finally, contentious areas, specifically anesthesia and animal preparations, should be approached jointly by multiple laboratories to ensure that the manipulations lead to reproducible results between centers, and to generate a nucleus around which a consensus can emerge. Such efforts will be necessary for the emergence of animal population imaging studies centered on brain function. Such efforts, likewise to human neuroimaging is expected to dramatically accelerate our understanding of the mammalian brain, its evolution, and the pathological mechanisms which affects its function.

## Author Contributions

JG designed the study and collected and processed the data. All authors contributed to the manuscript preparation.

## Conflict of Interest

The authors declare that the research was conducted in the absence of any commercial or financial relationships that could be construed as a potential conflict of interest.

## Footnotes

1 https://www.ncbi.nlm.nih.gov/pubmed/

2 https://central.xnat.org/

3 https://openneuro.org/
